# New insights in vitiligo: cellular immune response

**DOI:** 10.1186/1939-4551-8-S1-A137

**Published:** 2015-04-08

**Authors:** Natasha Ferraroni, Rosemeire Navickas Constantino-Silva, Camila Cacere, Juliano Cesar Barros, Rafael Aragon Cabrera, Anete Grumach, Carlos D'apparecida Santos Machado Filho

**Affiliations:** 1 Hospital De Base Do Distrito Federal, Brazil; 2 Laboratory of Clinical Immunology, Center of Research, Brazil; 3 Outpatient Group of Vitiligo, Brazil; 4 Outpatient Group of Recurrent Infections, Brazil

## Background

Vitiligo is a skin disorder that affects 1% to 2% of the world population, independently of ethnicity. It presents with white plaques and skin discoloration. The presence of antibodies against melanocytes confirms the autoimmune phenomena in this disease. Regarding cellular imune response in active vitiligo, it seems to be an imbalance between T cell CD8+ and CD4+, and, moreover, an altered expression of Natural Killer (NK) in periphery, although very few data are available. We evaluated the cellular immune effect (T cell expression) in peripheral blood in vitiligo patients who received antigenic stimulus (autologous graft), in comparison with patients with inactive Vitiligo who received autologous graft in comparison with patients with active Vitiligo without grafting.

## Methods

Antigenic stimulus was done with autologous skin graft (Punch 3mm): substitution of vitiligo area with normal skin in group A (inactive vitiligo patients), and group B (active vitiligo patients). Group C: healthy individuals. Quantitative numbers of T lymphocytes subpopulations (CD3, CD4, CD8) and NK cells (CD16, CD56, CD94, CD158a) were determined by Flow cytometry. (CD94+ refers as an inhibitor receptor expressed in NK cells, CD158+ refers as an apoptosis receptor in NK cells).

## Results

Three groups were evaluated: A) Inactive vitiligo patients engrafted (n=10); B) active vitiligo patients without engraftment (n=10) and C) healthy individuals (n=10). The evaluation was performed on days 0,+8,+30,+60 after skin engraftment. There was no difference of CD3+CD4+ among all groups. CD3+CD8+ was lower in patients with active vitiligo (p=0.003), CD94+ was lower in patients with inactive vitiligo (p=0.01), both comparing to healthy individuals. CD158+ was higher in patients with active vitiligo, although there was no statistically significance.

## Conclusions

Data suggests that cytotoxic activity of NK cells may be down regulated in patients with active vitiligo.

## Acknowledgements

Supported by FAPESP (Fundação de Amparo à Pesquisa do Estado de São Paulo).

